# New Elements of Operative Surgery

**Published:** 1846-07

**Authors:** 


ART. V.—*New Elements of Operative Surgery.* By ALF. A. L. M. VELPEAU: with a Treatise on Minor Surgery. Illustrated by 300 Engravings; and an Atlas in quarto of 22 Plates. First American, from the last Paris edition. Translated by P. S. Townsend, M. D.: Augmented by several hundred pages of new matter, under the supervision of, and *with Notes and Observations by Valentine Mott, M. D.* In three volumes, 8vo.—2d vol. Henry G. Langley, New-York, 1846.

"The second volume of this first American edition of Velpeau's Operative Surgery, as translated and with notes, by Dr. Mott and myself, has at length been completed, and is now offered to the profession."—p 1 of preface.

The *notes* only are announced as the joint labor of Drs. Mott and Townsend, and we congratulate all who feel a just pride in the reputation of American Surgeons, since Dr. Mott's natural and well known "repugnance to self laudation" would have forbidden any allusions to himself, that the duty of writing the preface has providentially fallen to his "kinsman" Dr. Townsend.

"I feel constrained also to add, that the accomplishment of this part of my labor, is one to which considerations of love of country add in my mind no slight degree of value. For young as our country is in history, and filled as its annals are with great names, both in the heroic period of its settlement, and in its subsequent ages of military renown, national emancipation, and diffusion of the arts, there is in my humble judgment no name that adorns those annals either in the battle field, or in the councils of government, or in its diplomacy, that has added more sterling reputation, and abiding lustre to the intrinsic glory and future fame of America than that of VALENTINE MOTT, unaided and ungilded though that name may be by the insignia of office or of power.

"For one who in his duties as a christian and a man of science, has done so much personally in the active field of benevolence, and struck out such noble discoveries and new paths to enable us more effectually to alleviate the miseries of our fellow creatures, there will be a balm as we believe, that will rest upon his memory in after time, less dazzling perhaps, but certainly full as enduring, and far more endearing to future generations, than any that the most ardent homage of patriotism could feel inspired with, for the glare of the most brilliant civic or military exploits.

"Thus much have I deemed it my duty here to record personally, at least of the views entertained by myself of the appreciation due to the accomplished Surgeon with whom I have had the honor to be associated in these labors."—*P. v. of preface.*

The first eighty pages of this volume, is a "supplemental appendix," comprising numerous extracts from the late Medical Journals, with some original communications to the translator, a portion of which matter might, perhaps, properly enough, claim a place in a work of this character, but we doubt the propriety of inserting in an elementary work for students, all the ill-digested and more than doubtful novelties of the science, encumbering the book and the mind of the pupil with a mass of speculation which a few years or months will determine to have been nothing but useless lumber. It certainly, however, helps very much to fill out the book to the proposed dimensions, and to such as do not take any of the standard Medical Journals, we recommend its perusal. We come now to the body of the work, and under the title "Traumatic Lesions of large Arterial Trunks, Direct Compression," &c., we read from the pen of Dr. Townsend as follows:

"Dr. Mott believes he has seen an instance lately, in which the subclavian artery, without the scaleni muscles, may have been wounded during an operation for the removal of a small tumour above the clavicle. The wound was probably of the nature of a *small flap* on its superior surface, between the first rib and the scalenus anticus muscle. In fact, the artery was perhaps nicked in this part, as a terrific hemorrhage ensued."

Which "flap," we are informed, was closed, and the hemorrhage both temporarily and permanently arrested by the application of numerous small and well adapted pieces of compressed sponge.

This we understand to be a reference to the unfortunate case of Mrs. B. of New-York, who was operated upon by Dr. S. P. White for the removal of a "small subcutaneous tumor" above the clavicle, and which having been attended with an unexpected hemorrhage and followed by death at the expiration of about four weeks, has since occasioned no inconsiderable discussion.

Wholly removed, as we certainly are by our remote situation, and by our lack of personal acquaintance with either of the contending parties from any special interest in their respective fates, we shall claim for our opinion, after a rigid examination of the whole matter, entire impartiality and honesty. And in speaking of the case, we affirm that we have but one object, namely, to prevent, as far as possible, a "great case," reported by high authority, from doing a great injury. Whether, therefore, the lady died finally of a "salivation," a "constipation" or a "sponge," we shall not debate; but whether Dr. Mott closed up a wound of the subclavian by a method at once most simple, novel and marvellous, now that it has been recorded by the Dr. himself, it is proposed to enquire. We shall also take the liberty, since our space is limited, to state our belief, and our reasons therefor, in the briefest possible manner, referring our readers to the book before us, and to the mutual publications of the belligerent parties for a more extended knowledge of the merits of the controversy.

We believe, then, *that the subclavian artery was not cut at all*, not even "nicked;" and of course that this (so called by Dr. Mott) "great case," and "first case" of closing a flap of a large artery by sponge, has not yet occurred.

Because, first, the size and situation of the tumor forbid the supposition that the subclavian could have been wounded in its removal. It was, says Dr. Cox, in his pamphlet *versus* Dr. White, "a small subcutaneous tumor" on "the shoulder;" others declare, and it is not denied, that it was about the size of a hickory nut, and well on to the shoulder. Now we know nothing about Dr. White's dexterity as a surgeon, nor do we care to know, for we affirm that no man ever had the dexterity to extirpate such a tumor, thus situated, and at the same moment wound the subclavian, as Mott says was probably done, "between the first rib and the scalenus anticus muscle" and just at the point "where it emerges from the scalenus;" and we are astonished beyond measure, that any man, either anatomist or practical surgeon, should ever have given currency to such an idea.

Because, second, the blood did not flow *per saltum*, but "welled up."

Because, third, the hemorrhage was at first and for two hours arrested by a slight syncope and moderate pressure.

[We omit to make use of Dr. White's statement as to the small amount of blood which was lost, since this point is in dispute, and we choose to appeal only to what is not denied. That the syncope was but slight is conceded.]

Because, fourth, when the hemorrhage returned it was first arrested by pressure with the finger, while the pressure "did not arrest the radial pulsation."

Because, fifth, it was finally and permanently arrested by small pieces of sponge used as a compress and without obliterating or even temporarily closing the channel of the artery!

In addition to which, we make the negative argument that the subclavian artery, it is not pretended, was either seen or felt as the source of the hemorrhage—no critical examination of the wound was made—and no one has described the depth of the wound as being any thing more than superficial until a hemorrhage had already occurred under sutures and a compress, within a shut cavity and to such an extent, as every one familiar with the loose character of the cellular connections in this region and with the usual effect of such shut up effusions, must see was sufficient to have increased very much the actual depth of the wound. The fingers, also, of Drs. Cox, Post and White, were consecutively pressed firmly into the wound to arrest the bleeding before Dr. Mott arrived, and clearly such pressure was enough to dissect quite down to the subclavian, and to carry into the very bottom of this now deep wound the bleeding vessel, whether vein or artery. How, then, we ask, could Dr. Mott determine by simply putting a finger into the channel that a "small flap" had been made in the subclavian? or rather, what right, from all this, had he even to *suppose* such an occurrence?

But we say farther and positively, that if the artery had been so "nicked," no such means could ever have stayed for one moment the fatal result. And this is precisely the practical point upon which we wish to be understood, lest we be thought to give sanction to a most dangerous error—dangerous certainty, if hereafter the practice should be considered as authority when the subclavian, or any other artery of equal size, is actually "nicked." The size of the artery, its proximity to the heart, and especially the character of its middle coat, which is thicker and denser as you approach the heart, and which M. Velpeau himself says prevents the wounds or *incomplete* divisions of the larger trunks from cicatrizing by agglutination, (p. 2 of second vol.) all forbid the possibility of reuniting such a "little flap" or "nick" by the above means, and in the manner related. As soon would we think of closing a "flap" in the dykes of the Mississippi by a cotton bag, as of restoring a breach in the walls of the subclavian by a sponge, and we feel bound to say, with Dr. White, that when Dr. Mott asserted, ex cathedra, that Dr. White had "nicked" the subclavian, he "nicked" his reputation, and in a manner most unkind and unfair. In our opinion, the distinguished operator will gain more honor by a successful application of the "sponge" and a complete restoration of this latter wound, than by any publication of his triumph in the first.

The radical cure of aneurisms, by compression, applied to the main trunk which supplies the tumor, as lately revived and improved by the surgeons of Dublin, is now attracting a large share of the attention of operators, and Dr. Townsend has very judiciously devoted several pages to a complete review of the subject. He concludes by remarking:

"The two great principles then of this *cure of aneurism by compression*, as laid down by Dr. Bellingham, and which are undoubtedly the only rational grounds by which we can hope for success, resolve themselves into these:—

1. "*Alternate Compression on different parts of the sound portions of the track of the Artery on the cardial side of the tumour*, by means of the ingenious and simple compressors now used with such unvarying success by the Dublin surgeons.

2. "*The diminution of the volume of blood in the affected trunk and not its total interruption.*—Of these two fundamental axioms, however, it must be borne in mind, that the latter which is of infinitely greater pathological importance of the two, and necessarily the result of the mechanical means of compression employed in the first, is not as might at first be inferred a new principle, but was well known to those Surgeons who advocated and made trial of compression in the early part of the present century. Various instruments, after extensivly detaching the femoral, were then tried, for pressing upon the vessel, *without impeding circulation through the limb*, and with success in cases of popliteal aneurism."

This mode of treating aneurisms, although we do not think it is destined, to any great extent, to supersede the Hunterian ligature or the ligature of Anel, as Velpeau prefers to designate it, still we cannot agree with Mr. Symes of Edinburgh, that it must soon return to its original obscurity. The practice already numbers at least thirteen completely successful cases, some of which were of a very unpromising character for any operation, and what with its present success, its admitted simplicity, safety and other occasional advantages, it will doubtless hereafter be frequently resorted to by prudent and enlightened surgeons.

We are pleased to find in this volume one long chapter on the subject of aneurisms, from the pen of Dr. Mott himself, and no one can read it attentively without feeling a sincere regret that upon this subject alone he has given us the benefit of his valuable experience; and it furnishes, we think, very good evidence of the correctness of our taste, when we would have Dr. Mott write down for us his own thoughts, rather than that another should write them for him. The whole chapter is so eminently practical and judicious that we wish we had space for all, but since we have not, we must be pardoned for presenting portions which strike us as possessing most general interest, although in a mutilated and disconnected form.

"More difficulty attends the diagnosis of aneurisms throughout the chest and lower part of the neck, than practitioners who are merely theoretically acquainted with the subject can possibly be aware of. All the additional light that has been thrown on the subject by auscultation will be admitted by all practical men, to be as yet an insufficient guide. Many cases are clear and obvious; others on the contrary, are obscure, and remain unknown until autopsic examinations disclose the truth, when it is too late for the interposition of therapeutic means."

This remark is illustrated by a case of aneurism of the aorta, which Dr. Mott himself and others, declared after a careful examination, assisted by the exploring needle and the stethoscope in the hands of a skillful stehoscopist, to be a malignant tumor, and the actual state of the case was not revealed until after death.

"We think there cannot be a better established principle than that the energies of the system frequently require aid, in order to enable it to remove surgical diseases. The extravagant, depletory, and starving system of Valsalva, in aneurisms, and of other practitioners for other diseases, deserve to fall, as they are doing rapidly, into disrepute."

"We have had some experience also in wounds of *the brachial artery in venesection.* We are happy to say that in our long career of practice, we have never had the misfortune to wound this artery with the lancet, but we have several times had occasion to serve our neighbors in this calamity. The first and paramount thing to be recollected whenever this accident should befall any person, is to compress the brachial artery somewhere in its course above the wound, and *never to attempt* compression at *the point wounded.* For no compression that can be made by an ordinary person will prevent the extravasation of blood."

"If a *false aneurism* shall have *formed simply between the artery and the vein*, our practice always would be, to tie the brachial somewhere in its course above, and leave the aneurismal tumour untouched. If the *vein be involved with the circumscribed aneurism*, the more secure practice certainly is, to tie the brachial artery above and below the aneurism, and exsect the sac; but in every case I would prefer the more simple practice of tying the brachial somewhere above, and leaving the diseased parts untouched; hoping that the resourses of nature would lead to a successful result. If they did not, the former or more severe operation must be resorted to.

"*If the artery should be wounded, and transmit its blood directly into the vein, the former vesssel healing securely and firmly to the under surface of*

*the vein, and only pouring a small quantity of blood directly into it, and thereby distending it an inch or two above and below the cicatrix in the vein*, our observation and experience lead us to say, that *nothing is to be done*.

"We have not observed even any weakness in such arms; and persons accustomed to laborious employments may be assured that generally no such consequences results from it.

"We have seen one *true* aneurism on the *ulnar artery* in its lower third. Whenever an aneurism shall be seated in either of the arteries of the fore-arm, the *radial* or *ulnar* ought always to be tied below the elbow if there is room enough. If not, the brachial must be resorted to.

"We have known of several instances of aneurisms in the *palm of the hand* from punctured wounds. The first and very natural step for a surgeon to take, is to compress the arteries at the wrist, one after the other, to determine from which palmar arch the aneurism proceeds. Most generally it will be from the superficial palmar; and therefore on compressing the trunk of the ulnar, the pulsation in the aneurismal tumour will cease. We would recommend, however, that both radical and ulnar arteries be tied in every such case, in order to render the cure certain.

"It will not be amiss in this place to state that in all wounds in the palm of the hand in which the branches divided cannot readily be discovered and tied, it is better in all cases to tie both arteries at the wrist rather than be satisfied with the one only whose compression at the wrist shall appear to stop the hemorrhage. We have, in a number of instances from our own experience, seen the hemorrhage return after a number of days; indeed even when the wound was granulating, say nine or ten days after the accident, making it then necessary to resort at last to the second artery of the wrist where only one at first had been tied."

"My reason for urging that both arteries should be tied at once, even though one should command the hemorrhage, is this, that by tying both simultaneously, you give time for the wounded artery to heal before a free inosculation can be established in the hand, and thereby revive the hemorrhage.

"By tying one after the other with an interval of some days between, you do not diminish sufficiently the inosculating circulation to prevent the recurrence of hemorrhage.

"It may not be amiss for me to connect with this important surgical subject, the fact that I have succeeded with *compressed sponge* in these wounds, where I formerly was in the habit of tying the arteries. The sponge ought always to be cut into small pieces, as it is in this way more readily introduced into the bottom of the wound, and by successive pieces makes more complete pressure in all parts of it, and possesses in an eminent degree the great advantage, afterwards, of being gradually removed, as the suppurating process comes on, without doing violence to, or lacerating the newly united vessels. For when a single large piece only is introduced, a very considerable force is afterwards neccessary to detach it, which thereby endangers a return of the hemorrhage."

"We would advise all who tie large arteries, to bear in mind, that after the edge of a muscle is laid bare, which is the anatomical guide or landmark for the relative situation of the artery, that very little use should be made of the knife.

"With his fingers, or the handle of the scalpel, the surgeon can readily separate the parts, so as fully to expose the artery. In this way he will be much less troubled with the oozing of blood, from cutting the small vessels, and thereby better enabled to see the principal trunk more distinctly.

"With the parts held asunder with curved spatulas, the surgeon now seizes the filamentous structure with the forceps, and raises it from the artery. He then cautiously divides the structure *perpendicularly, and upon the anterior surface of the artery only, and should never dissect or use the edge of the knife on the sides of the artery, but introduce the handle of the knife, and separate the structure from the artery on each side, only denuding the vessel to an extent barely sufficient to allow the hook to be passed around it.*

"This rule we believe most important, as by using the edge of the knife on the sides of the artery we endanger frequently the division of branches, as most of these are given off laterally; and the flow of blood where they are divided, obscures and interferes very much with the beauty and the neatness of the operation.

"Denuding the artery, also, to any considerable extent of its filamentous structure must, by robbing the vessel of its connecting media, always be adverse to the salutary changes which we expect from the ligature."

"My *mode of dressing the wound after tying the femoral artery*, is to pass a single stitch through the integuments in the centre of the wound. Short straps of adhesive plaster then answer to bring the remainder of the lips into contact.

"I then wrap the whole limb in wadding of wool; place the patient in bed, with the limb a little flexed and turned a little outward, with a pillow under the ham.

"No bandage of any sort is to be applied on any account whatever. We even avoid *long pieces* of adhesive plaster, for fear that by their compression the inosculating circulation might be interupted.

"Nothing is more dangerous than the application of a tight bandage to an aneurismal limb after the artery is tied: as everything that interferes with the collateral circulation must be to the greatest degree hazardous."

Section sixth, devoted to operations upon the venous system, as also section seventh, devoted to the nervous system, are replete with valuable instructions, and will be read with interest by every surgeon.

Under the subject of amputation, we find the following important lesson:

"The phrase *white swelling* is, moreover, one of too vague an import, at the present day, to have any value as an indication of amputation, (Jeanselme, *Arch. Gen. de Med.*, 1837.) It is upon the character of the disease and of the tissues affected, and not from the title of white swelling, that we are to make up our judgment upon the propriety of amputation in diseases of the joints, (arthropathies.) If the capsule has been for a long time filled with pus: if there are fistulas existing about the joints, and the friction made on the surfaces leave no doubt as to the extent of the necrosis or caries; if, also, the ligaments and surrounding fibrous layers are destroyed, and an ichorous fluid escapes in large quantities, and a fungoid or fatty degeneration has involved the synovial membrane and most of the soft tissues; if the limb be atrophied both above and below, and is luxated, or has a tendency to become so; if, in a word, it is manifest that the bones and the cartilages have been for a long time the seat of a deep-seated, destructive alteration in the parts; then is amputation indicated: though the cure, even where all this mischief exists, does sometimes ultimately take place in the articulations, especially in those of the fingers."

The section comprises all that is necessary to be said upon this important branch of surgery, and is, in itself, a volume, extending through more than 200 pages.

Under the section devoted to the subject of exsections, we find the following remark by M. Velpeau, in relation to what is called in this country, "Physic's Mode" of treating ununited fracture, viz: by the seton.

"It is nevertheless a very uncertain means, and one for which I should prefer to substitute exsection, where frictions and the starch bandage had not answered, and it is one moreover which is not always unattended with danger. M. Weinhold in applying it to the neck of the femur brought on caries and suppuration in the cotyloid cavity and pelvis, which ended in the death of his patient."

Exsection, also, in similar cases, M. Gouraud considers a retrograde movement in surgery. The other methods employed are friction, rasping, cauterization, compression, and immoveable apparatus. Dr. Mott has treated several by the seton successfully; and one case is reported as cured by exsection; but he lays it down as a rule that unless the bones are in actual contact the seton cannot be relied upon, and that the more the ends of the bone ride over each other the better is the prospect of success. When the bones thus ride, Dr. Mott, says Dr. Townsend, has occasionally found it necessary to drill a hole through the ligamentous tissue existing between their surfaces before passing the seton.

On page 882 will be found a review of Dr. Mott's claim to priority in "exsection of the lower jaw;" but we do not think Dr. Townsend establishes any thing farther than the justness of his claim to priority in the "exsection of a *portion* of the lower jaw for *osteo sarcoma*, which operation was first made by Dr. Mott in 1821. M. Dupuytren excised a portion of the inferior maxilla for a "carcinome" in 1812, and many other operators had previously made similar exsections for various diseases of the jaw. To the same conclusion, also, the committee of the New-York Medical Society seem to have arrived, who made their investigation and report in 1830. If, however, as has been said, M. Dupuytren laid claim to priority in the much more formidable operation of exsection of the lower jaw for "osteo sarcoma," he certainly, and his countrymen have, thus far, wholly failed to support the claim. To Dr. Mott, and through him to our country, does the honor of the first attempt and successful completion of this surgical achievement belong. The strictures upon the remarks of Dr. Houston, of Dublin, who ascribes the first suggestion to his friend Mr. Cusach, are merited, if Mr. Houston *knew* the facts; if he did not, he deserves at least reproof for his ignorance.

Mr. Liston says, in his lectures, that the operation of removing the clavicle for fibro-cartilaginous tumors is attended with "some little difficulty," &c. Dr. Townsend, in the name of Dr. Mott, regards the remark as conveying a very pernicious error, since Dr. Mott believes this operation "one of the most dangerous and difficult, if not *the most so*, of any to which the human body has ever been subjected." Dr. Mott has made the operation himself, and has long been in the practice of distinguishing it as his "Waterloo Operation," which soubriquet is in itself sufficiently indicative of the light in which he regards it—it is the the triumph of a Wellington over a Bonaparte! And instead of the humble clavicle from the museum of the Meckels, which, so far as we know, is the only monument erected in commemoration of the deed, it deserves, if Dr. Mott is right, a memento as lofty and enduring as the stupendous triumphal mound and colossal bronze lion, which now overlook the bloody plains of St. Jean.

Following a section upon the use of the trephine, the volume before us closes with the subject of tumors; in each of which divisions we have marked much practical and highly interesting matter, but the extent to which we have already extended this review, compels us to refer the reader to the book itself. F. H. H.

